# Comprehensive analyses of glycolysis-related lncRNAs for ovarian cancer patients

**DOI:** 10.1186/s13048-021-00881-2

**Published:** 2021-09-24

**Authors:** Jianfeng Zheng, Jialu Guo, Linling Zhu, Ying Zhou, Jinyi Tong

**Affiliations:** 1 Department of Obstetrics and Gynecology, Affiliated Hangzhou Hospital, Nanjing Medical University, Hangzhou, 310008 Zhejiang Province China; 2 Department of Obstetrics and Gynecology, Hangzhou Women’s Hospital, No.369 Kunpeng Road, Shangcheng District, Hangzhou, 310008 Zhejiang Province China; 3 Department of Fourth Clinical Medical College, Zhejiang Chinese Medical University, Hangzhou, 310006 Zhejiang Province China; 4 Department of Clinical Pharmacology, Key Laboratory of Clinical Cancer Pharmacology and Toxicology Research of Zhejiang Province, Affiliated Hangzhou First People’s Hospital, Zhejiang University School of Medicine, Hangzhou, 310008 Zhejiang Province China

**Keywords:** Ovarian cancer, Glycolysis, lncRNA, Risk model, Immune

## Abstract

**Background:**

Not only glycolysis but also lncRNAs play a significant role in the growth, proliferation, invasion and metastasis of of ovarian cancer (OC). However, researches about glycolysis -related lncRNAs (GRLs) remain unclear in OC. Herein, we first constructed a GRL-based risk model for patients with OC.

**Methods:**

The processed RNA sequencing (RNA-seq) profiles with clinicopathological data were downloaded from TCGA and glycolysis-related genes (GRGs) were obtained from MSigDB. Pearson correlation coefficient between glycolysis-related genes (GRGs) and annotated lncRNAs (|r| > 0.4 and *p* < 0.05) were calculated to identify GRLs. After screening prognostic GRLs, a risk model based on five GRLs was constructed using Univariate and Cox regression. The identified risk model was validated by two validation sets. Further, the differences in clinicopathology, biological function, hypoxia score, immune microenvironment, immune checkpoint, immune checkpoint blockade, chemotherapy drug sensitivity, N6-methyladenosine (m6A) regulators, and ferroptosis-related genes between risk groups were explored by abundant algorithms. Finally, we established networks based on co-expression, ceRNA, cis and trans interaction.

**Results:**

A total of 535 GRLs were gained and 35 GRLs with significant prognostic value were identified. The prognostic signature containing five GRLs was constructed and validated and can predict prognosis. The nomogram proved the accuracy of the model for predicting prognosis. After computing hypoxia score of each sample by ssGSEA, we found patients with higher risk scores exhibited higher hypoxia score and high hypoxia score was a risk factor. It was revealed that a total of 21 microenvironment cells (such as Central memory CD4 T cell, Neutrophil, Regulatory T cell and so on) and Stromal score had significant differences between the two groups. Four immune checkpoint genes (CD274, LAG3, VTCN1, and CD47) showed disparate expression levels in the two groups. Besides, 16 m6A regulators and 126 ferroptosis-related genes were expressed higher in the low-risk group. GSEA revealed that the risk groups were associated with tumor-related pathways. The two risk groups were confirmed to be sensitive to several chemotherapeutic agents and patients in the low-risk group were more sensitive to ICB therapy. The networks based on co-expression, ceRNA, cis and trans interaction provided insights into the regulatory mechanisms of GRLs.

**Conclusions:**

Our identified and validated risk model based on five GRLs is an independent prognostic factor for OC patients. Through comprehensive analyses, findings of our study uncovered potential biomarker and therapeutic target for the risk model based on the GRLs.

**Supplementary Information:**

The online version contains supplementary material available at 10.1186/s13048-021-00881-2.

## Introduction

Ovarian cancer (OC) is a gynecological tumor with high morbidity and mortality and about 150,000 women die of OC each year [1]. The occurrence and development of OC is a multi-system, multi-step cellular biochemical process, which is regulated by a variety of cytokines and signaling pathways [2]. Due to the lack of typical clinical symptoms in the early stages of OC, 75% of OC patients are diagnosed at an advanced stage, and more than 70% of patients relapse after treatment [3]. Therefore, how to diagnose early, effectively treat and improve the prognosis of OC patients is an urgent problem to be solved.

Tumor cells are mainly metabolized by glycolysis regardless of the presence of oxygen. Large amounts of glucose are consumed with the production of lactic acid. This phenomenon is called aerobic glycolysis or Warburg effect [4]. Long non-coding RNA (lncRNA) is defined as a large class of non- protein-coding, regulatory RNAs with molecules longer than 200 nucleotides, which play key roles in tumorigenesis and progression [5, 6]. In recent years, more and more studies have shown that lncRNA plays a key regulatory role in tumor metabolism and is involved in glucose metabolism pathway [7, 8]. For instance, lncRNA ANRIL up-regulates the expression of glucose transporter 1(GLUT1) and LDHA, thereby increasing glucose uptake and promoting the progression of nasopharyngeal carcinoma [9]. LINC00092 directly binds to PFKFB2 to enhance glycolysis and ultimately promote tumor genesis and development [10]. In bladder cancer, lncRNA UCA1 is overexpressed and promotes glycolysis by upregulation of hexokinase 2 (HK2), and also promotes aerobic glycolysis [11]. However, lncRNAs involved in the glycolysis reprogramming of OC remain unclear.

Therefore, in our study, we found five glycolysis-related lncRNAs (GRLs) with significant prognostic value from TCGA dataset. A GRL-signature with prognostic value was developed. In addition, we identified differences in enrichment pathways, immune microenvironment, immune checkpoints, m6A regulatory factors, and ferroptosis-related genes between risk groups. The networks based on co-expression, ceRNA, cis and trans interaction provided insights into the regulatory mechanisms of GRLs.

## Material and methods

### Data downloading and pretreatment

We downloaded the clinical data with RNA sequencing profiles of OC patients from TCGA dataset [12]. The Ensemble expression matrix was transformed into Gene Symbol expression matrix and compared it with the position of lncRNA chromosome in GENCODE database to identify lncRNAs [13]. A total of 274 glycolysis-related genes (GRGs) were obtained from MSigDB database [14]. We screened differentially expressed GRGs and annotated lncRNAs using limma package (*P* < 0.05, |logFC| > 1) [15]. Pearson correlation coefficients between differential GRGs and lncRNAs were computed to filtrate GRLs (|r| > 0.4, *P* < 0.05) using cor function of R.

### Development of the signature

After screening prognostic GRLs through Univariate Cox regression (*P* < 0.05) [16], the LASSO Cox regression [17] from glmnet package of R [18] and 20 times cross-validation analysis was employed to filtrate optimal combination of GRL markers. A risk score model for OC patients was constructed based on following formula:

$$\mathrm{Risk}\ \mathrm{score}=\sum {\upbeta}_{\mathrm{lncRNA}}\times {\mathrm{Exp}}_{\mathrm{lncRNA}}$$

In the risk score (RS) formula, β_lncRNA_ meant the regression coefficient of each lncRNA calculated in the multivariate Cox regression analysis and Exp_lncRNA_ represented the expression value of each lncRNA in the sample. Whereafter, the RS of each OC patient was calculated and the calculated median RS was used as the critical value to further divided the OC patients into high-risk and low-risk groups (low-risk group<median, high-risk group ≥ median). Furthermore, in order to verify the accuracy of the signature in predicting the prognosis, all samples (total set, TS) were randomly and evenly divided into two validation sets (VS1 and VS2).

We used the timeROC package in the R language to draw ROC curves to evaluate the predictive ability to predict 1, 2, 3, and 5 years of survival. Besides, a visual nomogram was constructed and verified by the calibration curve to determine the accuracy of the risk model for serving as an independent prognostic factor.

### Characteristics and application of the signature

To explore the relationship and degree of correlation between potential GRGs and biological pathways in OC, biofunctional analysis was performed on potential GRGs using DAVID [19, 20].

Considering that hypoxic microenvironment is closely related to aerobic glycolysis of OC, we downloaded a collection of hypoxia-related genes (HRGs), HALLMARK HYPOXIA, from MSigDB. The enrichment fraction of hypoxia pathway in different samples was calculated based on ssGSEA arithmetic from GSVA of R to obtain hypoxia scores [21]. Simultaneously, the expression levels of immune checkpoint genes (CD274, CD47, HAVCR2, LAG3, SIRPA, TNFRSF4 and VTCN1), m6A regulators [22], and ferroptosis-related genes (FRGs) [23] were extracted to contrast their expression differences in risk groups by intergroup T test.

The immune microenvironment is also closely related to the occurrence and development of OC. According to the expression data of the OC samples, the immune and stromal scores were estimated by ESTIMATE to represent the presence of stroma and immune cells [24]. Based on ssGSEA [25], the enrichment fraction of 28 immune cells was calculated to represent the relative abundance of each TME-infiltrated cell in OC samples. Moreover, three algorithms CIBERSORT [26, 27], MCPcounter [28], and xCell [29], were wielded to compare the difference in the proportion of various immune cells in different risk groups.

In order to explore the relevant regulatory mechanism of this risk prediction model, we established networks based on co-expression, ceRNA, cis and trans interaction. GRG-GRL co-expression (|r| > 0.4, *P* < 0.05) network was constructed and visualized by Cytoscape [30]. The targeted glycolysis-related mRNAs by corresponding miRNAs were speculated by miRWalk [31]. Further, we synthesized the results of six commonly used databases (miRWalk, Microt4, miRanda, miRDB, RNA22 and Targetscan) to obtain the miRNA-GRG relationship pair if the predicted miRNA-GRG relationship pair appeared in ≥5 databases. The miRNAs targeted by corresponding GRLs of the risk model were speculated by miranda (v3.3a, Score > =140, Energy<= − 20) [32]. GRLs and GRGs regulated by the same miRNA with positive co-expression relationship were defined as ceRNAs mutually. Based on previous literature, we predicted cis [33] and trans [34] interaction between GRLs and GRGs.

Moreover, the potential response to immune checkpoint blockade (ICB) was predicted with TIDE algorithm [35]. We extracted chemotherapy drugs from GDSC database [36] and evaluated the IC50 level by using pRRophetic [37].

### Statistical analysis

R packages (v4.0.2) and GraphPad Prism (v8.0) were used for statistical analysis. T test was used for inter-group comparison. Pearson correlation analysis was conducted to analyze the correlation between GRGs and lncRNAs. Univariate and multivariate Cox regression analysis was conducted to analyze the related factors affecting the overall survival of OC patients. *P* < 0.05 was considered statistically significant.

## Results

**Fig. 1:**
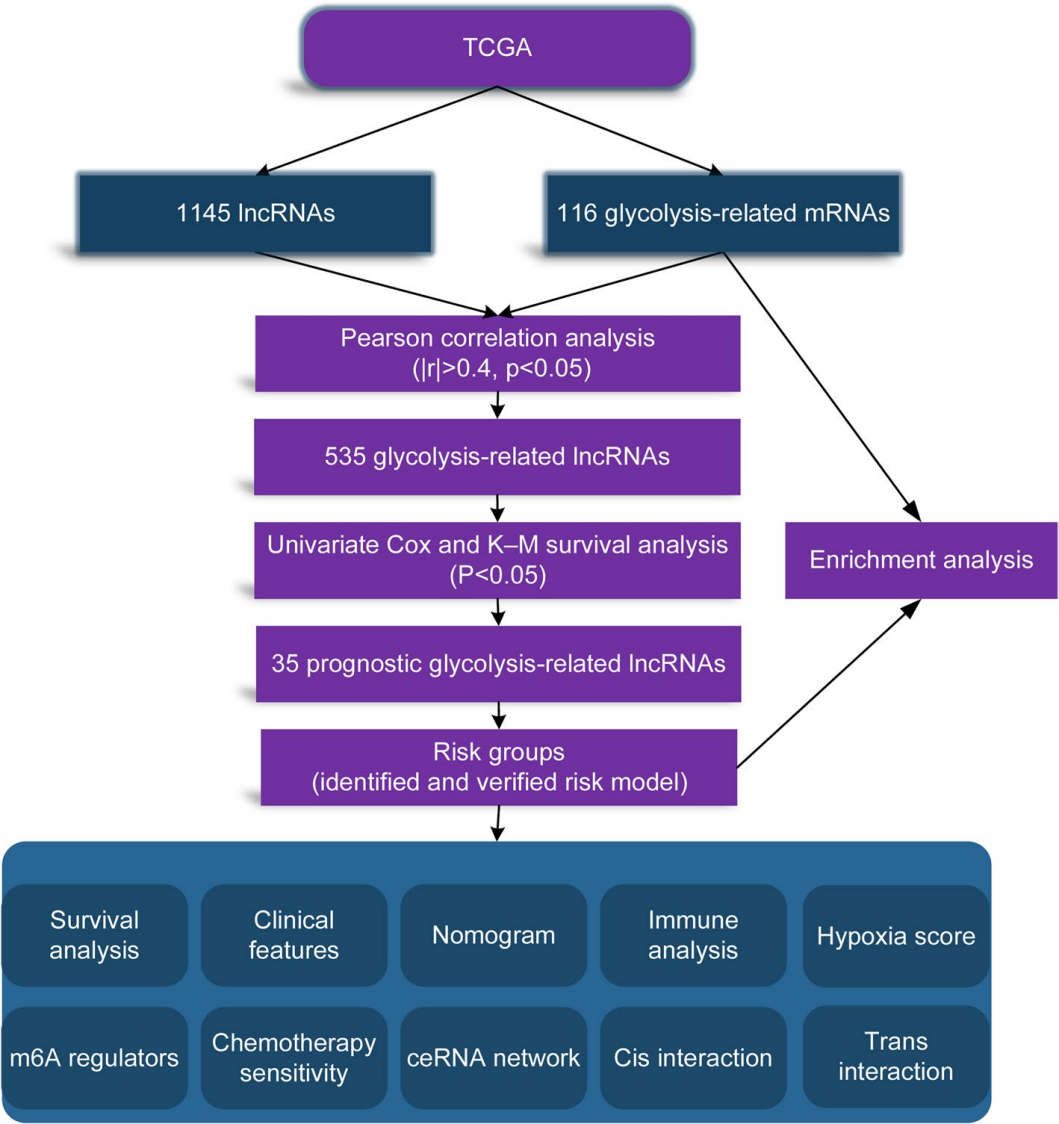
Flow diagram of our study

Figure 1 exhibited the flowchart we created for our entire study.

### Differential and enrichment analysis

**Fig. 2:**
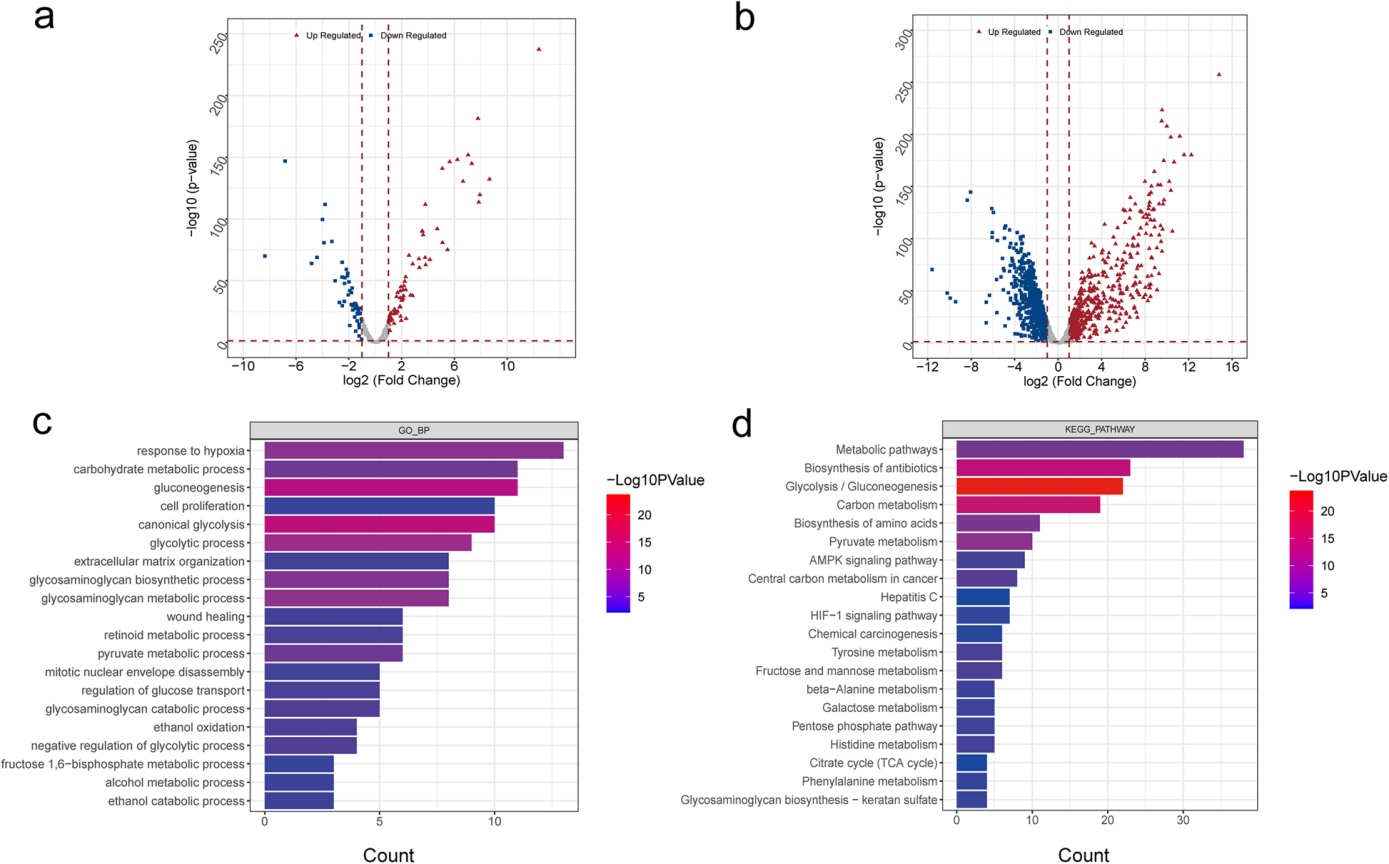
Differential and enrichment analysis. **A**, **B** Volcano map of differentially expressed mRNAs (**A**) and lncRNAs (**B**). Red triangle: up regulated; blue square: down regulated. **C** GO analysis. The color scale represented *p* value and the histogram size indicated count. **D** KEGG analysis. The color scale represented *p* value and the histogram size indicated count

A total of 116 differential GRGs (Fig. 2A) and 1145 exegetical lncRNAs (Fig. 2B) were identified. In addition, 62 GO BP and 33 KEGG pathways were enriched based on the 116 GRGs (Additional file 1: Table S1). The enrichment pathways were ranked according to *p* value, and the top 20 was selected for display (Fig. 2C-D). The result showed that most of these differential GRGs were enriched in metabolic pathways. In addition, the identified GRGs were associated with several important biological processes in tumor genesis and development observably, such as, response to hypoxia, AMPK signaling pathway, HIF-1 signaling pathway, and so on. This further proves that glycolysis is closely related to tumor hypoxic microenvironment.

### Construction and validation of the risk model based on GRLs

**Fig. 3:**
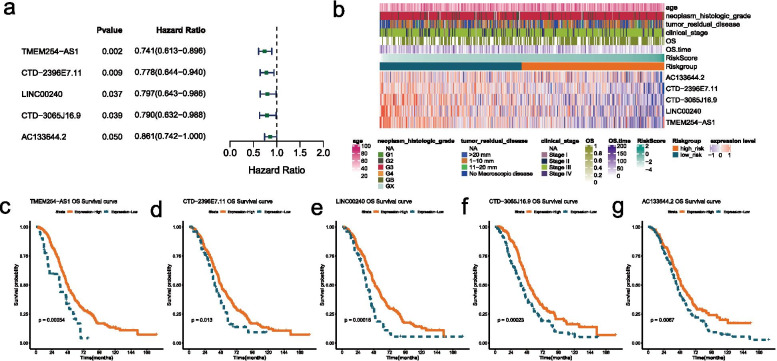
Features of the five GRLs. **A** Forest plot of the prognostic ability of the nine optimal GRLs. All the five GRLs were protective factors with HR < 1. **B** Heatmap of the associations between the expression levels of the five GRLs and clinical features. **C**-**G** The K-M survival curves of the five optimal GRLs. TMEM254 − AS1 (**C**), CTD − 2396E7.11 (**D**), LINC00240 (**E**), CTD − 3065 J16.9 (**F**), and AC133644.2 (**G**)

Univariate Cox regression and K–M survival analysis was performed on 535 GRLs acquired from Pearson correlation analysis (|r| > 0.4, *p* < 0.05) to excavate GRLs with significant prognosis (*P* < 0.05). A prognostic GRL-signature was constructed according to the LASSO Cox analysis of 35 prognostic GRLs obtained and a total of five GRLs were selected to build the risk model (Table 1). The results showed that all the five GRLs were protective factors with HR < 1 (Fig. 3A). A heatmap of the associations between the expression levels of the five GRLs and clinical features illustrated that the expression of the expressions of the five GRLs decreased with increasing risk scores (Fig. 3B). The K–M survival curves confirmed that higher expression of them were associated with better OS of OC patients (Fig. 3C-G).

**Table 1:** The coefficients of the five GRLs

**GRL**	**coef**	**logFC**
**AC133644.2**	−0.149267499	4.214519614
**CTD-2396E7.11**	−0.250907132	6.5602714
**CTD-3065 J16.9**	−0.235361863	1.732399016
**LINC00240**	−0.227486983	1.732645895
**TMEM254-AS1**	−0.2997883	−1.568258405

**Fig. 4:**
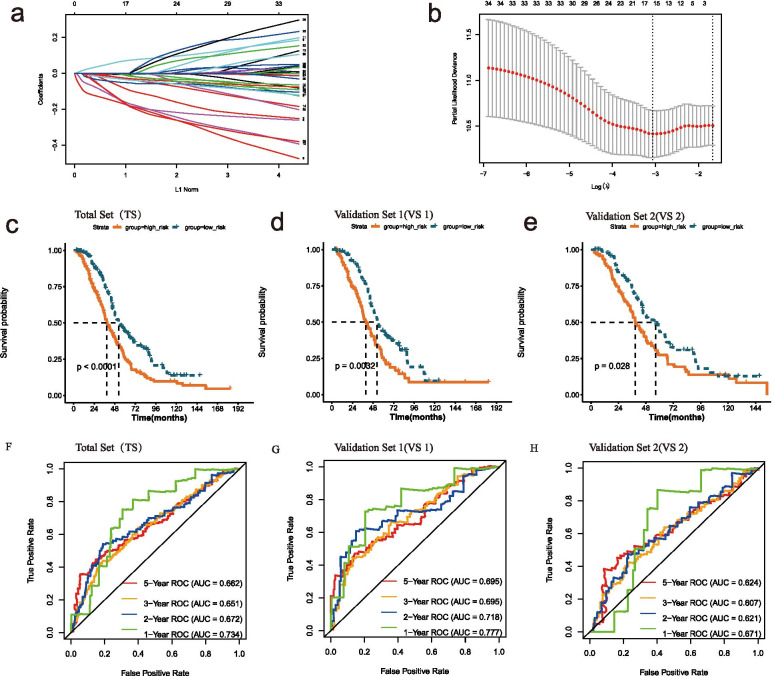
Construction and validation of the risk model. **A** LASSO Cox analysis. **B** λ selection diagram. The two dotted lines indicated two particular values of λ. The left side was λ_min_ and the right side was λ_1se_. The λ_min_ was selected to build the model for accuracy in our study. **C**-**E** The K-M survival curves of total (**C**) and validation sets (**D**, **E**). **F**-**H** Time-dependent ROC curve analysis of total (**F**) and validation sets (**G**, **H**)

The λ selection diagram was shown in Fig. 4A-B. The OC patients were divided into two risk subgroups based on the mean of RSs. The K–M survival curves revealed that OS of the high-risk group was markedly lower than that of the low-risk group in TS (Fig. 4C), VS1 (Fig. 4D), VS2 (Fig. 4E), which indicated the accuracy of the risk model in predicting survival status. The time-dependent ROC curve proved that the risk assessment model was relatively stable in predicting 1-year, 2-year, 3-year and 5-year survival for OC patients (The AUC for survival was over 0.6, Fig. 4F-H).

**Fig. 5:**
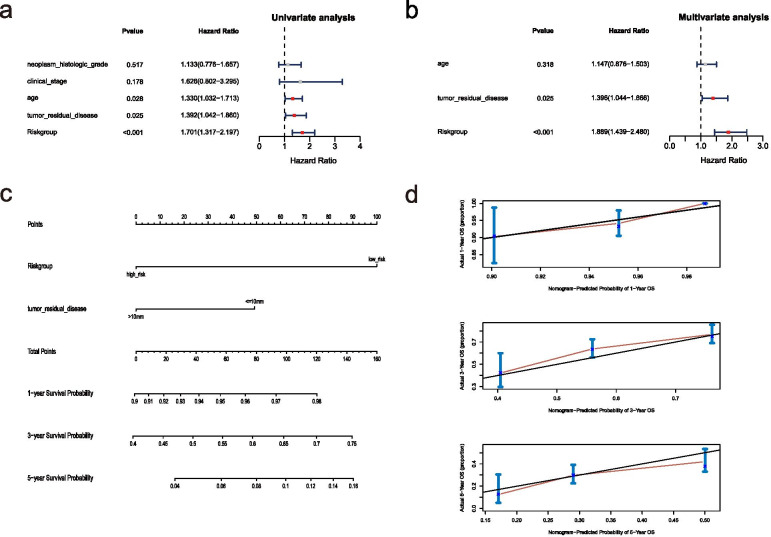
Nomogram construction. **A** The Univariate analysis of risk model and clinical features. **B** The Multivariate analysis of risk model and clinical features. **C** The Nomogram model based on risk model and clinical features. **D** The calibration plots of the nomogram. The closer it was to 45 degrees or the gray lines in the graph, the better the fitting effect

The univariate and multivariate Cox regression analysis of clinical features and the risk model demonstrated that “tumor residual disease” and risk model was an independent prognostic factor for OC patients (Fig. 5A-B). A nomogram was further constructed based on “tumor residual disease” and risk model (Fig. 5C). The calibration curve (the closer it was to 45 degrees or the gray lines in the graph, the better the fitting effect) was drawn to prove the accuracy of the model (Fig. 5D).

In conclusion, our risk model was a stable, independent prognostic factor for OC.

### Functional pathways of the risk groups

**Fig. 6:**
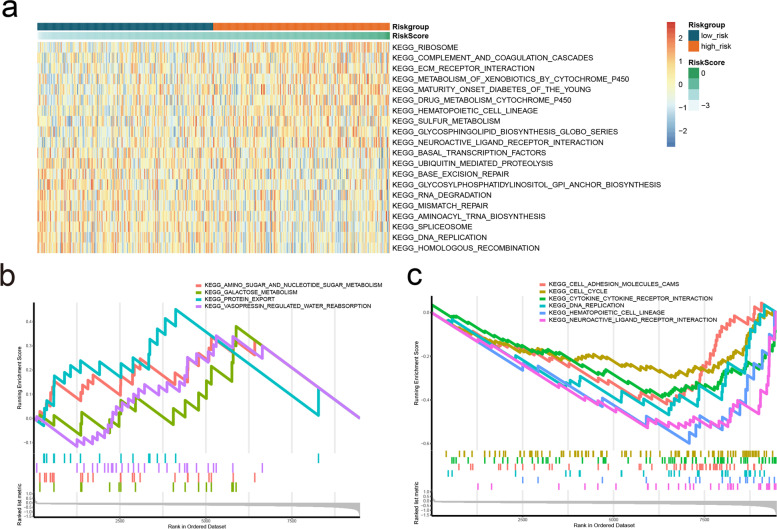
Differences in functional pathway between the risk groups. **A** Top10 KEGG Pathway GSVA enrichment score heat map. **B**-**C** The GSEA of KEGG pathway in the two risk groups. Significant enrichment in the high-risk group (**B**); Significant enrichment in the low-risk group (**C**)

Functional pathway enrichment analysis based on GSVA algorithm showed that a total of 66 pathways exhibited significant differences between the two risk subgroups (Additional file 2: Table S2). The KEGG pathways were ranked according to the *p* value, and the top ten were selected for display (Fig. 6A). According to GSEA enrichment analysis, four pathways were enriched in the high-risk group (Fig. 6B), and six pathways were enriched in the low-risk group (Fig. 6C).

### Hypoxia score analysis

**Fig. 7:**
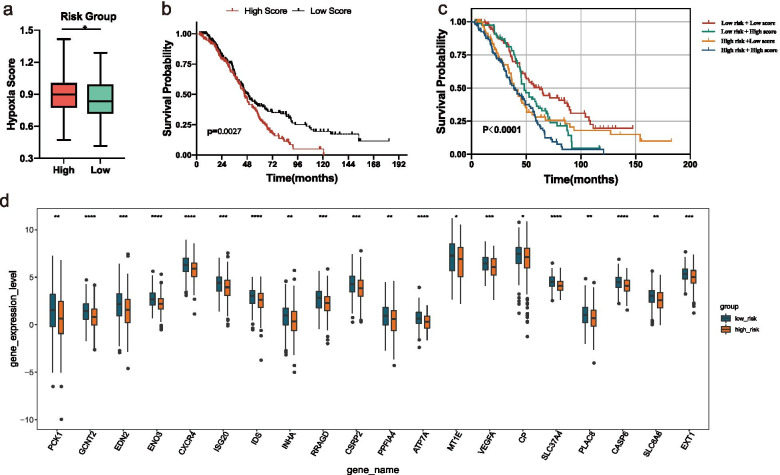
Hypoxia score analysis. **A** Hypoxia scores between high and low-risk groups. **B** The K–M survival curves of OC patients with high or low hypoxia score. **C** The K–M survival curves of four subgroups based on risk score and hypoxia score. **D** Top20 hypoxia-related gene expression distribution box diagram of the difference multiple between the high and low risk groups. *p < 0.05, **p < 0.01, ***p < 0.001, ****p < 0.0001

Considering that the aerobic glycolysis of tumor is closely related to its hypoxic microenvironment, the hypoxia enrichment score of each sample was calculated. Interestingly, we found that patients in the high-risk group had a higher hypoxia score (Fig. 7A). According to the median of hypoxia scores, the OC patients were divided into two subgroups. The K–M survival curves revealed that OS of the patients with high hypoxia score was markedly lower (Fig. 7B), indicating that high hypoxia score and high-risk score were both risk factors (Fig. 7C). Between the two risk groups, a total of 76 differential hypoxia-related genes were received (Additional file 3: Table S3) and the top 20 were displayed in Fig. 7D.

### Immunity microenvironment and checkpoint analyses

**Fig. 8:**
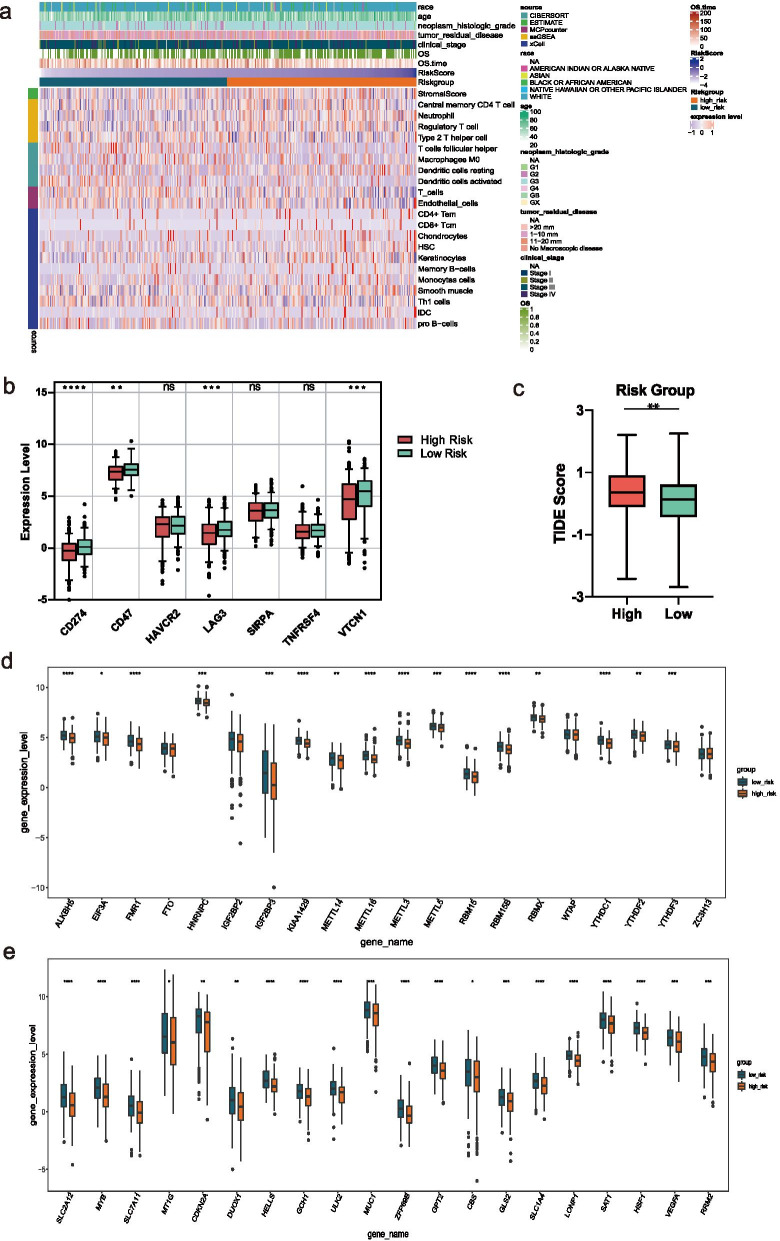
Immune and gene analysis. **A** Heatmap of immune microenvironment revealed that a total of 21 immune cells and stromal score had significant differences between the two risk groups. **B** Expression of seven immune checkpoint genes between high and low-risk group. CD274, LAG3, VTCN1, and CD47 had a lower expression in the high-risk group. **C** TIDE scores in the low-risk group were lower than those in the high-risk group. **D** The expression of 20 m6A regulators between high and low-risk groups. **E** The expression of top20 ferroptosis-related genes between high and low-risk groups. Data are shown as means ± S.D. ns: not significant, *p < 0.05, **p < 0.01, ***p < 0.001, ****p < 0.0001

We assessed immune status by applying five algorithms mentioned in the Methods section, which was shown after merging the five algorithms in the heat map (Additional file 4: Figure S1). Further, Wilcoxon was utilized to compare the significance of each cell between the two groups. Results showed that a total of 21 microenvironment cells and Stromal score emerged remarkable differences (Fig. 8A).

Immunosuppressive checkpoint inhibitors play a biological role by inhibiting the immunosuppressive signal pathway in the immune system. In view of this, in order to further explore the clinical application of the risk model, we compared the difference in seven checkpoint genes between the two risk groups. The expression distribution box diagram of the seven immune checkpoint genes (CD274, CD47, HAVCR2, LAG3, SIRPA, TNFRSF4, and VTCN1) between the two risk groups was shown in Fig. 8B. The results showed that CD274, LAG3, VTCN1, and CD47 had a lower expression in the high-risk group. The TIDE score was correlated closely with response to immune checkpoint blockade (ICB). In Fig. 8C, OC patients in low-risk group exhibited lower TIDE scores than those in high-risk group, indicating that OC patients with lower RSs were more sensitive to ICB therapy.

### m6A and ferroptosis analyses

The expression levels of m6A regulators and FRGs between the two risk groups were also compared. A total of 20 m6A regulators were matched, and as can be seen, except for FTO, IGF2BP2, WTAP, and ZC3H13, the expression levels of the remaining 16 m6A regulators were significantly higher in the low-risk group (Fig. 8D). A total of 126 FRGs were matched and showed significant differences between the high and low-risk groups (Additional file 5: Table S4). The top20 genes ranked by the difference multiple were shown in Fig. 8E. It can be seen that all the genes had low expression in the high-risk group.

### Sensitivity of chemotherapy drug

**Fig. 9:**
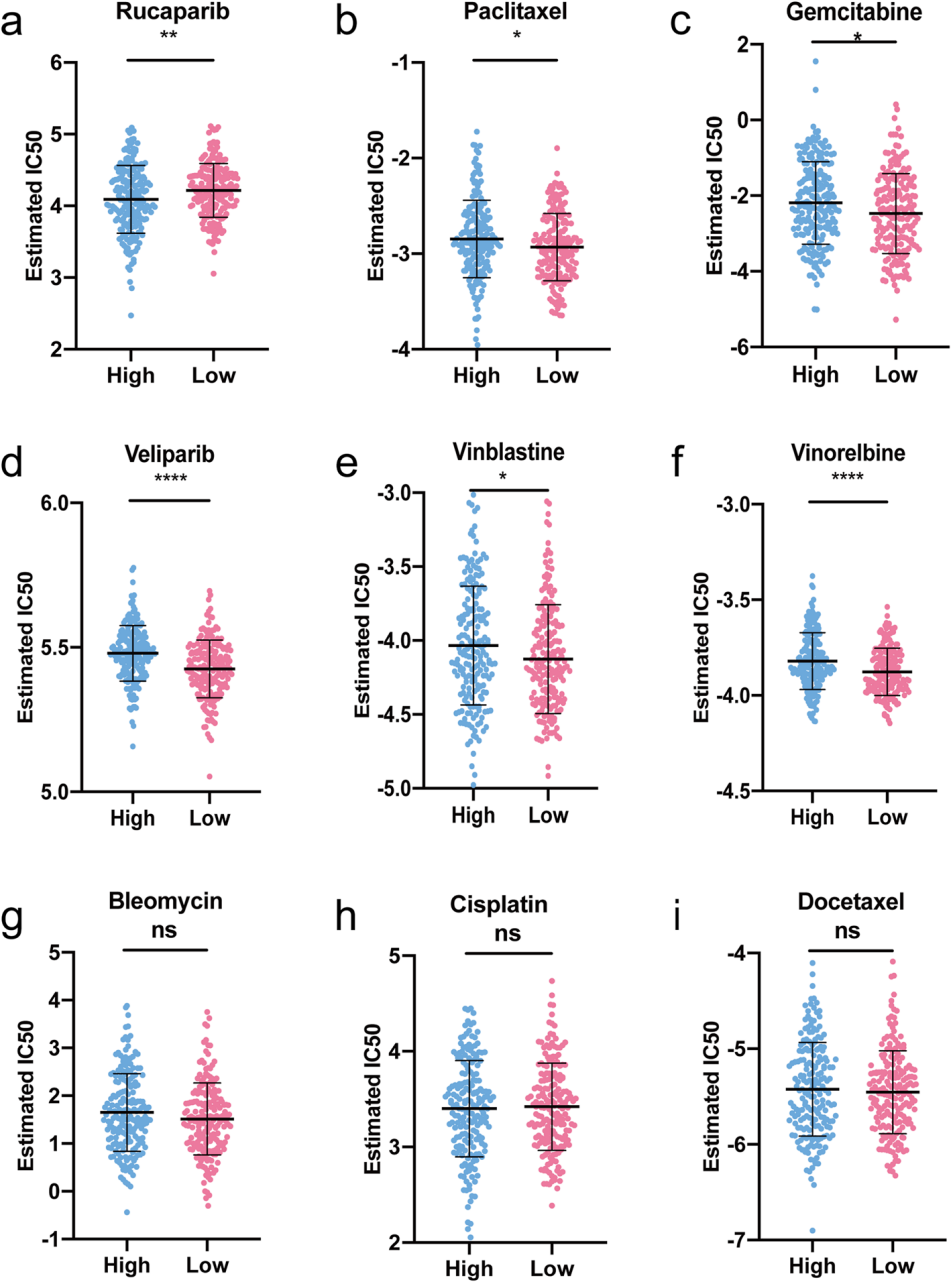
Sensitivity of chemotherapy drugs. **A**-**I** Difference in the estimated IC50 levels of Rucaparib (**A**), Paclitaxel (**B**), Gemcitabine (**C**), Veliparib (**D**), Vinblastine (**E**), Vinorelbine (**F**), Bleomycin (**G**), Cisplatin (**H**), and Docetaxel (**I**). Data are shown as means ± S.D. ns: not significant, *p < 0.05, **p < 0.01, ***p < 0.001, ****p < 0.0001

In light of the significance of chemotherapy in the treatment of OC, we quantified the response ability of OC patients with different risk scores to 137 chemotherapeutic drugs. We compared IC50 values for nine commonly used chemotherapeutic agents in two risk groups (Fig. 9). A lower IC50 value indicated that this group of patients was more sensitive to the drug. Our data showed that the IC50 levels of Rucaparib (Fig. 9A) were significantly higher in the low-risk group than that in high-risk group. Inversely, the IC50 levels of Paclitaxel (Fig. 9B), Gemcitabine (Fig. 9C), Veliparib (Fig. 9D), Vinblastine (Fig. 9E), and Vinorelbine (Fig. 9F) were significantly lower in low-risk group than that in high-risk group, indicating that the OC patients in the low-risk group were more sensitive to these drugs. However, the sensitivity of the two risk groups to Bleomycin (Fig. 9G), Cisplatin (Fig. 9H), and Docetaxel (Fig. 9I) did not reach significant difference.

### Network analyses

**Fig. 10:**
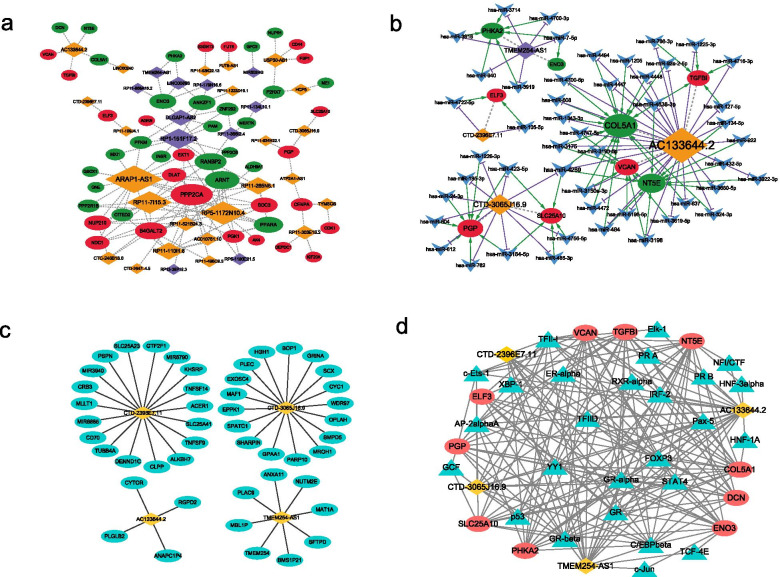
Network analyses. **A** GRG-GRL co-expression network. Ellipses: GRGs; rhombus: GRLs. **B** ceRNA network. Ellipses: GRGs; rhombus: GRLs; arrowhead: miRNAs. **C** lncRNA-nearby mRNA interaction networks. Rhombus: GRLs; ellipses: nearby mRNAs. **D** Trans interaction for lncRNA-TF-mRNA relationship pairs. Rhombus: GRLs; ellipses: mRNAs; triangle: TFs

In order to explore the relevant regulatory mechanism of this risk prediction model, we established networks based on co-expression, ceRNA, cis and trans interaction. A total of 3524 GRG-GRL co-expression relation pairs were obtained ((|r| > 0.4, *p* < 0.05) (Additional file 6: Table S5). Only 35 GRLs with significant prognosis and the corresponding co-expression pair network were selected to fabricate the GRG-GRL co-expression network (Fig. 10A). A total of 59 GRL-miRNA-GRG relationship pairs were obtained, including four GRLs, 48 miRNAs, and nine GRGs (Additional file 7: Table S6, Fig. 10B). The nine GRGs included: COL5A1, ELF3, ENO3, NT5E, PGP, PHKA2, SLC25A10, TGFBI, and VCAN. The networks based on the cis and trans interaction were displayed in Fig. 10C-D. Interestingly, we found that p53 may regulate GRLs and GRGs through trans interactions. The regulatory relationships revealed by these networks may provide a direction for exploring the molecular mechanism of the GRLs.

## Discussion

The fate of tumor cells is directly related to their energy metabolism [38]. Tumor cells prefer glycolysis as an inefficient metabolic mode, which provides new ideas and methods for clinical treatment of tumors [39]. The reasons are as follows [40]: glycolysis can provide the energy needed for tumor cell proliferation; It can maintain a low pH tumor microenvironment, which is conducive to tumor cell proliferation, drug resistance, invasion and metastasis; A large number of nucleic acid precursors can be produced in preparation for proliferation. For ovarian cancer and other tumors with high proliferation, invasion, metastasis and chemotherapy resistance, it is of great significance to explore the regulation of its glycolytic pathway. The aim of studying the glycolysis pathway of OC is to develop ideal targeted drugs. However, the mechanism of action of some drugs targeting the glycolytic pathway of OC is still not clear, so the in-depth study of their molecular mechanism is still of great significance.

As is known to all that lncRNAs have been proved to play an important role in the occurrence and development of tumors. In recent years, lncRNAs have been reported to regulate the energy metabolism of tumors and thus affect the malignant behavior of tumors, which also partially reveals the molecular mechanism of glycolysis reprogramming. For example, lncRNA AGPG increases the stability of PFKFB3 by inhibiting ubiquitination at Lys302 and subsequent proteasomal-dependent degradation of PFKFB3 and activates glycolytic flux, causing metabolic reprogramming in esophageal cancer cells [41]. In addition, PFKFB3 can also be phosphorylated by lncRNA YIYA, increasing the conversion of fructuce-6-phosphate to fructuce-2, 6-Bisphosphate, and promoting the reprogramming and growth of glucose metabolism in breast cancer [42]. However, researches of GRLs are still scarce in OC.

In order to verify the importance of glycolysis-related lncRNAs (GRLs) in ovarian cancer progress, GRL-related prognostic and diagnostic model were developed. The gene expression level of 535 GRLs were in investigated in OC and normal tissues. The significance of these GRLs related to survival rates were then studied and 35 GRLs were discovered significantly prognostic. In our study, we identified and validated a signature containing five GRLs with prognostic value. A total of 21 microenvironment cells, four immune checkpoint genes (CD274, LAG3, VTCN1, and CD47), 16 m6A regulators, and 126 FRGs showed different levels between the two groups. It has been reported that the hypoxic and acidic microenvironment induced by tumor glycolysis can cause metabolization-mediated T cell dysfunction, which may be one of the mechanisms of tumor cell metabolic reprogramming mediated immune escape [43, 44]. It has also been found glycolysis of tumors can induce tumor immunosuppression and immune escape [45]. Therefore, tumor immunotherapy strategies based on metabolic regulation can improve the effectiveness of immunotherapy [45]. Many studies have found that m6A regulatory factors can regulate the expression of enzymes related to the glucose metabolism pathway, thus affecting the glycolysis of tumors [46–48]. All these provide a reference for us to study the specific mechanism of glycolysis in tumor. The occurrence and development of malignant tumors is sophisticated and we hope to explore the molecular mechanism of glycolysis (via m6A modification, ferroptosis or immune) to promote the efficacy of immunotherapy with further research.

Our study still has some limitations. Firstly, due to the limited number of OC samples that can annotate lncRNA expression, more patients with homologous information were needed to incorporate into study and prove the credibility of our study. Secondly, we explored the functions of these five lncRNAs only through bioinformatics analysis, and experimental data were needed to support these conclusions. Despite these limitations, our study used two validation sets, ROC, and nomogram to demonstrate the effectiveness of the risk model for prognostic prediction.

## Conclusions

In summary, our identified and validated risk model based on five glycolysis -related lncRNAs is an independent prognostic factor for OC patients. Through comprehensive analyses, the GRL-model provides insights into clinical applications for OC.

## Supplementary Information

**Additional file 1: Table S1.** The enriched 62 GO BP and 33 KEGG pathways based on 116 GRGs.

**Additional file 2: Table S2.** A total of 66 pathways exhibited significant differences between the two risk subgroups.

**Additional file 3: Table S3.** A total of 76 differential hypoxia-related genes were received.

**Additional file 4: Figure S1.** Immunity microenvironment analysis.

**Additional file 5: Table S4.** A total of 126 FRGs were matched and showed significant differences between the high and low-risk groups.

**Additional file 6: Table S5.** 3524 GRG-GRL co-expression relation pairs.

**Additional file 7: Table S6.** ceRNA.

## Data Availability

The RNA sequencing profiles are able to be gained from The Cancer Genome Atlas (TCGA) (https://toil.xenahubs.net). Further inquiries can be directed to the corresponding author.

## Abbreviations

OC: Ovarian cancer
GRL: Glycolysis-related lncRNA
TCGA: The Cancer Genome Atlas
GRG: Glycolysis-related gene
PCC: Pearson correlation coefficient
LASSO: Least absolute shrinkage and selection operator
m6A: N6-methyladenosine
ICB: Immune checkpoint blockade
GSEA: Gene Set Enrichment Analysis
RS: Risk score
OS: Overall survival
GRCh38: Genome Reference Consortium Human Build 38
HR: Hazard ratio
K-M: Kaplan–Meier
GO: Gene ontology

## References

[1] Lheureux S, Gourley C, Vergote I, Oza AM (2019). Epithelial ovarian cancer. Lancet (London, England).

[2] Narod S (2016). Can advanced-stage ovarian cancer be cured?. Nat Rev Clin Oncol.

[3] Siegel RL, Miller KD, Jemal A (2020). Cancer statistics, 2020. CA Cancer J Clin.

[4] Koppenol WH, Bounds PL, Dang CV (2011). Otto Warburg’s contributions to current concepts of cancer metabolism. Nat Rev Cancer.

[5] Wang JY, Lu AQ, Chen LJ (2019). LncRNAs in ovarian cancer. Clin Chim Acta.

[6] Braga EA, Fridman MV, Moscovtsev AA, Filippova EA, Dmitriev AA, Kushlinskii NE (2020). LncRNAs in ovarian cancer progression, metastasis, and main pathways: ceRNA and alternative mechanisms. Int J Mol Sci.

[7] Fan C, Tang Y, Wang J, Xiong F, Guo C, Wang Y, Zhang S, Gong Z, Wei F, Yang L (2017). Role of long non-coding RNAs in glucose metabolism in cancer. Mol Cancer.

[8] Xu Y, Qiu M, Shen M, Dong S, Ye G, Shi X, et al. The emerging regulatory roles of long non-coding RNAs implicated in cancer metabolism. Mol Ther. 2021;29(7):2209–18. 10.1016/j.ymthe.2021.03.017 PMC8261164 33775912

[9] Zou ZW, Ma C, Medoro L, Chen L, Wang B, Gupta R, Liu T, Yang XZ, Chen TT, Wang RZ (2016). LncRNA ANRIL is up-regulated in nasopharyngeal carcinoma and promotes the cancer progression via increasing proliferation, reprograming cell glucose metabolism and inducing side-population stem-like cancer cells. Oncotarget.

[10] Zhao L, Ji G, Le X, Wang C, Xu L, Feng M, Zhang Y, Yang H, Xuan Y, Yang Y (2017). Long noncoding RNA LINC00092 acts in cancer-associated fibroblasts to drive glycolysis and progression of ovarian cancer. Cancer Res.

[11] Li Z, Li X, Wu S, Xue M, Chen W (2014). Long non-coding RNA UCA1 promotes glycolysis by upregulating hexokinase 2 through the mTOR-STAT3/microRNA143 pathway. Cancer Sci.

[12] Goldman MJ, Craft B, Hastie M, Repečka K, McDade F, Kamath A, Banerjee A, Luo Y, Rogers D, Brooks AN (2020). Visualizing and interpreting cancer genomics data via the Xena platform. Nat Biotechnol.

[13] Harrow J, Frankish A, Gonzalez JM, Tapanari E, Diekhans M, Kokocinski F, Aken BL, Barrell D, Zadissa A, Searle S (2012). GENCODE: the reference human genome annotation for the ENCODE project. Genome Res.

[14] Liberzon A, Subramanian A, Pinchback R, Thorvaldsdóttir H, Tamayo P, Mesirov JP (2011). Molecular signatures database (MSigDB) 3.0. Bioinformatics (Oxford, England).

[15] Ritchie ME, Phipson B, Wu D, Hu Y, Law CW, Shi W, Smyth GK (2015). limma powers differential expression analyses for RNA-sequencing and microarray studies. Nucleic Acids Res.

[16] Wang P, Wang Y, Hang B, Zou X, Mao JH (2016). A novel gene expression-based prognostic scoring system to predict survival in gastric cancer. Oncotarget.

[17] Tibshirani R (1997). The lasso method for variable selection in the Cox model. Stat Med.

[18] Engebretsen S, Bohlin J (2019). Statistical predictions with glmnet. Clin Epigenetics.

[19] Huang DW, Sherman BT, Lempicki RA (2009). Systematic and integrative analysis of large gene lists using DAVID bioinformatics resources. Nat Protoc.

[20] Huang DW, Sherman BT, Lempicki RA (2009). Bioinformatics enrichment tools: paths toward the comprehensive functional analysis of large gene lists. Nucleic Acids Res.

[21] Hänzelmann S, Castelo R, Guinney J (2013). GSVA: gene set variation analysis for microarray and RNA-seq data. BMC Bioinformatics.

[22] Xu S, Tang L, Dai G, Luo C, Liu Z (2020). Expression of m6A regulators correlated with immune microenvironment predicts therapeutic efficacy and prognosis in gliomas. Front Cell Dev Biol.

[23] Zhou N, Bao J (2020). FerrDb: a manually curated resource for regulators and markers of ferroptosis and ferroptosis-disease associations. Database.

[24] Yoshihara K, Shahmoradgoli M, Martínez E, Vegesna R, Kim H, Torres-Garcia W, Treviño V, Shen H, Laird PW, Levine DA (2013). Inferring tumour purity and stromal and immune cell admixture from expression data. Nat Commun.

[25] Jia Q, Wu W, Wang Y, Alexander PB, Sun C, Gong Z, Cheng JN, Sun H, Guan Y, Xia X (2018). Local mutational diversity drives intratumoral immune heterogeneity in non-small cell lung cancer. Nat Commun.

[26] Newman AM, Liu CL, Green MR, Gentles AJ, Feng W, Xu Y, Hoang CD, Diehn M, Alizadeh AA (2015). Robust enumeration of cell subsets from tissue expression profiles. Nat Methods.

[27] Charoentong P, Finotello F, Angelova M, Mayer C, Efremova M, Rieder D, Hackl H, Trajanoski Z (2017). Pan-cancer Immunogenomic analyses reveal genotype-immunophenotype relationships and predictors of response to checkpoint blockade. Cell Rep.

[28] Shi J, Jiang D, Yang S, Zhang X, Wang J, Liu Y, Sun Y, Lu Y, Yang K (2020). LPAR1, correlated with immune infiltrates, is a potential prognostic biomarker in prostate cancer. Front Oncol.

[29] Aran D, Hu Z, Butte AJ (2017). xCell: digitally portraying the tissue cellular heterogeneity landscape. Genome Biol.

[30] Shannon P, Markiel A, Ozier O, Baliga NS, Wang JT, Ramage D, Amin N, Schwikowski B, Ideker T (2003). Cytoscape: a software environment for integrated models of biomolecular interaction networks. Genome Res.

[31] Dweep H, Gretz N (2015). miRWalk2.0: a comprehensive atlas of microRNA-target interactions. Nat Methods.

[32] Enright AJ, John B, Gaul U, Tuschl T, Sander C, Marks DS (2003). MicroRNA targets in drosophila. Genome Biol.

[33] Yuan CL, Jiang XM, Yi Y, Jian-Fei E, Zhang ND, Luo X, Zou N, Wei W, Liu YY (2019). Identification of differentially expressed lncRNAs and mRNAs in luminal-B breast cancer by RNA-sequencing. BMC Cancer.

[34] Shu X, Shu S, Cheng H (2019). A novel lncRNA-mediated trans-regulatory mechanism in the development of cleft palate in mouse. Mol Genet Genomic Med.

[35] Fu J, Li K, Zhang W, Wan C, Zhang J, Jiang P, Liu XS (2020). Large-scale public data reuse to model immunotherapy response and resistance. Genome Med.

[36] Yang W, Soares J, Greninger P, Edelman EJ, Lightfoot H, Forbes S, Bindal N, Beare D, Smith JA, Thompson IR (2013). Genomics of Drug Sensitivity in Cancer (GDSC): a resource for therapeutic biomarker discovery in cancer cells. Nucleic Acids Res.

[37] Geeleher P, Cox N, Huang RS (2014). pRRophetic: an R package for prediction of clinical chemotherapeutic response from tumor gene expression levels. PLoS One.

[38] Vander Heiden MG, DeBerardinis RJ (2017). Understanding the intersections between metabolism and cancer biology. Cell.

[39] Ganapathy-Kanniappan S, Geschwind JF (2013). Tumor glycolysis as a target for cancer therapy: progress and prospects. Mol Cancer.

[40] Vander Heiden MG, Cantley LC, Thompson CB (2009). Understanding the Warburg effect: the metabolic requirements of cell proliferation. Science (New York, NY).

[41] Liu J, Liu ZX, Wu QN, Lu YX, Wong CW, Miao L, Wang Y, Wang Z, Jin Y, He MM (2020). Long noncoding RNA AGPG regulates PFKFB3-mediated tumor glycolytic reprogramming. Nat Commun.

[42] Xing Z, Zhang Y, Liang K, Yan L, Xiang Y, Li C, Hu Q, Jin F, Putluri V, Putluri N (2018). Expression of long noncoding RNA YIYA promotes glycolysis in breast cancer. Cancer Res.

[43] Chang CH, Qiu J, O'Sullivan D, Buck MD, Noguchi T, Curtis JD, Chen Q, Gindin M, Gubin MM, van der Windt GJ (2015). Metabolic competition in the tumor microenvironment is a driver of cancer progression. Cell.

[44] Hatfield SM, Kjaergaard J, Lukashev D, Schreiber TH, Belikoff B, Abbott R, Sethumadhavan S, Philbrook P, Ko K, Cannici R (2015). Immunological mechanisms of the antitumor effects of supplemental oxygenation. Sci Transl Med.

[45] Jung KH, LoRusso P, Burris H, Gordon M, Bang YJ, Hellmann MD, Cervantes A, Ochoa de Olza M, Marabelle A, Hodi FS (2019). Phase I study of the Indoleamine 2,3-dioxygenase 1 (IDO1) inhibitor navoximod (GDC-0919) administered with PD-L1 inhibitor (Atezolizumab) in advanced solid tumors. Clin Cancer Res.

[46] Yu H, Yang X, Tang J, Si S, Zhou Z, Lu J, Han J, Yuan B, Wu Q, Lu Q (2021). ALKBH5 inhibited cell proliferation and sensitized bladder cancer cells to cisplatin by m6A-CK2α-mediated glycolysis. Mol Ther Nucleic Acids.

[47] Li Z, Peng Y, Li J, Chen Z, Chen F, Tu J, Lin S, Wang H (2020). N(6)-methyladenosine regulates glycolysis of cancer cells through PDK4. Nat Commun.

[48] Shen C, Xuan B, Yan T, Ma Y, Xu P, Tian X, Zhang X, Cao Y, Ma D (2020). Zhu X et al: m(6)A-dependent glycolysis enhances colorectal cancer progression. Mol Cancer.

