# Supplementary figures and images for: Comprehensive analyses of glycolysis-related lncRNAs for ovarian cancer patients
Source: J Ovarian Res. 2021 Sep 24;14:124. doi: 10.1186/s13048-021-00881-2 (PMC8464158; doi:10.1186/s13048-021-00881-2)

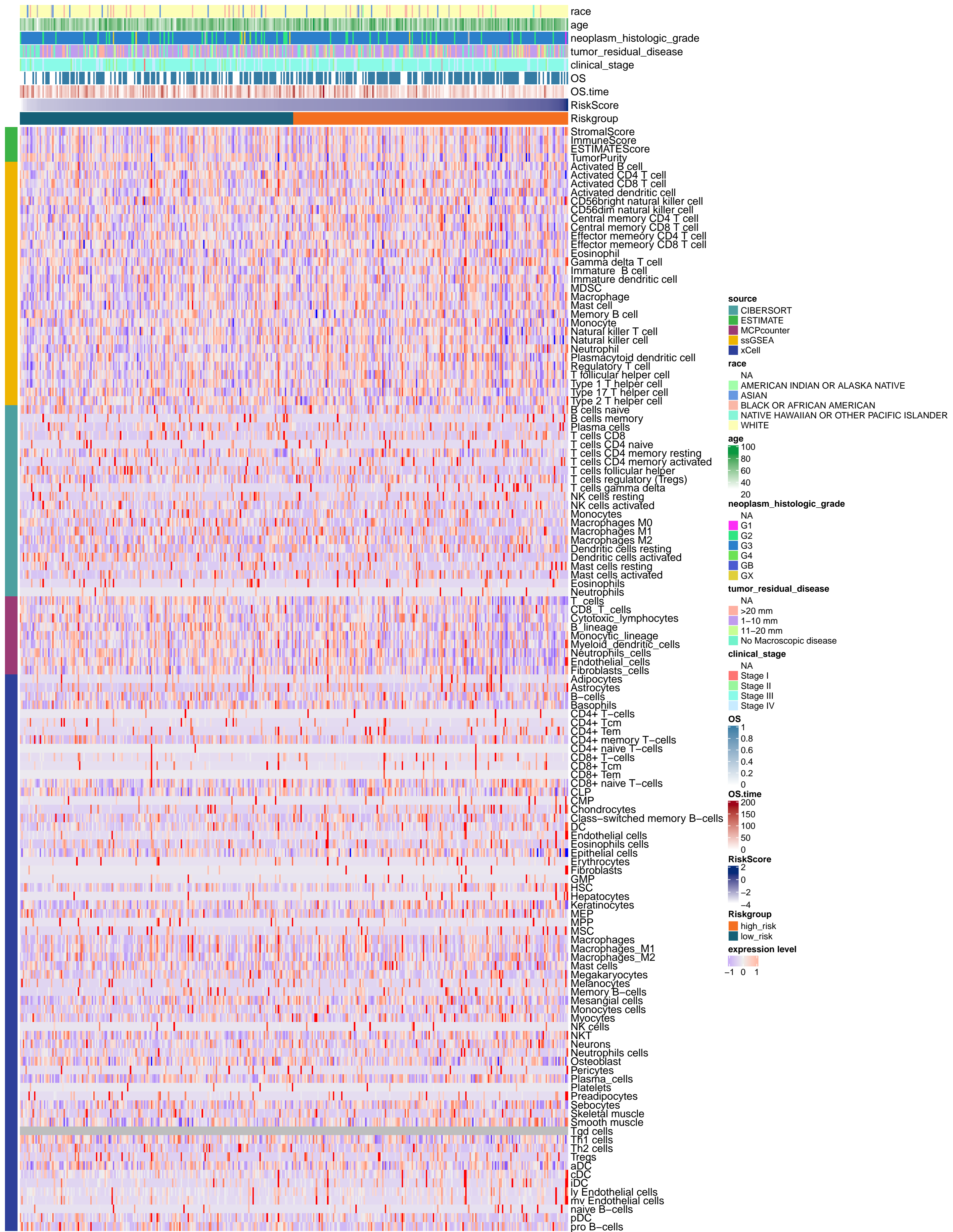

Supplement: Supplementary file 4 — Additional file 4: Figure S1. Immunity microenvironment analysis. [file 13048_2021_881_MOESM4_ESM.pdf]
